# Mixed-Mode Mindfulness-based cognitive therapy for psychological resilience, Self Esteem and Stigma of patients with schizophrenia: a randomized controlled trial

**DOI:** 10.1186/s12888-024-05636-z

**Published:** 2024-03-04

**Authors:** Jiali Dai, Dawei Sun, Bohui Li, Yang Zhang, Meiling Wen, Haina Wang, Hongsheng Bi

**Affiliations:** 1 Department of geriatric Psychiatry, The Third Hospital of Daqing, Daqing, China; 2 Clinical laboratory, The Third Hospital of Daqing, Daqing, China; 3 Second Psychiatric Ward, The Third Hospital of Daqing, Daqing, China; 4 Department of Psychological counseling, The Third Hospital of Daqing, Daqing, China; 5 Second Psychiatric Ward, the Third Hospital of Daqing, 163712 Daqing, China

**Keywords:** Mindfulness-based cognitive therapy, Psychological resilience, Self-esteem, Stigma, Schizophrenia

## Abstract

**Background:**

People with schizophrenia often face challenges such as lower psychological resilience, reduced self-worth, and increased social stigma, hindering their recovery. Mindfulness-Based Cognitive Therapy (MBCT) has shown promise in boosting psychological resilience and self-esteem while diminishing stigma. However, MBCT demands professional involvement and substantial expenses, adding to the workload of professionals and the financial strain on patients. Mixed-mode Mindfulness-Based Cognitive Therapy (M-MBCT) integrates both “face-to-face” and “self-help” approaches to minimize staff effort and costs. This study aims to assess the impact of M-MBCT on the psychological resilience, self-esteem, and stigma in schizophrenia patients.

**Methods:**

This randomized, controlled, parallel-group, assessor-blinded clinical trial enrolled 174 inpatients with schizophrenia. Participants were randomly assigned to either the experimental or control group. The experimental group underwent an 8-week M-MBCT intervention, while the control group received standard treatment. Data collection employed the Connor-Davidson Resilience Scale (CD-RISC), Internalized Stigma of Mental Illness Scale (ISMI), and Rosenberg Self-Esteem Scale (RSES) before and after the intervention. Post-intervention, significant differences in ISMI, CD-RISC, and RSES scores were observed between the experimental and control groups.

**Results:**

In the experimental group, ISMI scores notably decreased, while CD-RISC and RSES scores significantly increased (*P* < 0.05). Multiple linear regression analysis identified age, education, and family history of mental illness as significant factors related to stigma (*P* < 0.05). Additionally, correlation analysis indicated a significant negative relationship between the reduction in CD-RISC scores and the reduction in ISMI scores (*P* < 0.05).

**Conclusion:**

M-MBCT effectively enhanced psychological resilience and self-esteem while diminishing stigma in individuals with schizophrenia. M-MBCT emerges as a promising treatment option for schizophrenia sufferers.

**Trial registration:**

The trial was registered at the Chinese Clinical Trial Registry on 03/06/2023 (www.chictr.org.cn; ChiCTR ID: ChiCTR2300069071).

## Background

Schizophrenia is recognized as one of the most severe mental disorders, characterized by cognitive, emotional, and behavioral abnormalities, along with impaired social functioning [1, 2]. Currently, the primary approach for treating schizophrenia involves antipsychotic drugs, with most of these medications acting as dopamine receptor antagonists. Despite positive outcomes, many individuals do not achieve full recovery, and a diagnosis of schizophrenia profoundly impacts their lives, leading to social isolation and stigma [2, 3]. The use of antipsychotic drugs contributes to both direct stigma (e.g., being labeled as “crazy”) and indirect stigma (e.g., facing ridicule for weight gain), significantly affecting the adherence of individuals diagnosed with schizophrenia [3]. Patients with schizophrenia are particularly susceptible to self-stigmatization when confronted with societal stigma. Consequently, individuals with schizophrenia may internalize the prevailing negative judgments of society, leading to social isolation [4, 5], diminished self-esteem, and ultimately hindering rehabilitation [6]. Studies indicate a negative correlation between stigma and self-esteem [7, 8]. Stigma can contribute to depression, social withdrawal, reduced self-esteem, and heightened psychiatric symptoms in patients with schizophrenia [9, 10]. In the face of stigma, patients may refuse assistance and specialized treatment, causing them to miss optimal treatment opportunities. Hence, there is a pressing need for scientific and systematic psychological interventions to reduce the stigma associated with schizophrenia [5].

Patients with schizophrenia often exhibit low psychological resilience, a crucial mechanism enabling individuals to cope with adverse stressors by dynamically enhancing their adaptability through reshaping their relationship with the environment [11, 12]. The stigma experienced by schizophrenia patients is inversely related to their psychological resilience [13]. Enhanced psychological resilience correlates significantly with various aspects of illness progression (e.g., shorter duration of untreated psychosis, prolonged illness duration, improved symptom remission and recovery), internal factors (e.g., lower stigma, enhanced self-esteem), and psychosocial functioning (better overall, real-life, social, and interpersonal functioning, higher quality of life) [14]. Psychological resilience also serves as a mediator in pathways leading to depression, functioning, and quality of life within schizophrenia spectrum conditions [14, 15]. Elevating the psychological resilience of schizophrenia patients can mitigate stigma, enhance self-esteem, and promote overall disease recovery [14, 15].

Mindfulness promotes detached, non-judgmental observation of thoughts, perceptions, sensations, and emotions, enabling self-monitoring and arousal regulation through detached awareness. Mindfulness-Based Stress Reduction (MBSR) has demonstrated success in alleviating anxiety, chronic pain, fibromyalgia, mood, and stress, fostering an increased sense of control, particularly in individuals with cancer [16, 17]. Mindfulness-Based Cognitive Therapy (MBCT) integrates elements from mindfulness-based and cognitive behavior therapy, initially designed as a preventive and therapeutic measure for individuals prone to depression relapse, but later adapted for various populations and environments [18, 19].

MBCT has the potential to diminish stigma and facilitate the comprehensive rehabilitation of schizophrenia patients [20]. Concurrently, MBCT can ameliorate the mood of family members of schizophrenia patients by enhancing their psychological resilience [21]. However, traditional MBCT entails professional investment and high costs, elevating the workload for professionals and the economic burden on patients. Advancements in science and technology have enabled the exploration of more convenient self-help MBCT courses, shifting away from traditional face-to-face interactions. While online self-help mindfulness-based interventions have shown health benefits [22, 23], they are limited by the absence of supervision, therapist participation, and group discussions. Mixed-mode mindfulness-based courses, combining “face-to-face” group therapy and “self-help” elements, provide enhanced convenience, cost-effectiveness, and reduced personnel investments compared to traditional courses. In contrast to self-help interventions, mixed-mode courses effectively address limitations such as the lack of supervision, therapist involvement, and group discussions. Therefore, it is crucial to explore the impact of Mixed-mode Mindfulness-Based Cognitive Therapy (M-MBCT) on the psychological resilience, self-esteem, and stigma experienced by schizophrenia patients.

## Methods

The study strictly adhered to CONSORT guidelines [24].

### Study design and setting

To evaluate the impact of Mixed-mode Mindfulness-Based Cognitive Therapy on the resilience, self-esteem, and stigma of patients with schizophrenia, we conducted an 8-week, randomized, controlled, parallel-group, assessor-blinded clinical trial. Participants were recruited from the Inpatient Department of the Third Hospital of Daqing between January 2023 and July 2023. This hospital is one of the largest psychiatric facilities in Heilongjiang Province, China, accommodating approximately 1000 beds.

Inclusion Criteria: (a) Age 18–60. (b) Diagnosis of schizophrenia based on the International Classification of Diseases 10th Edition (ICD-10) Diagnostic Criteria. (c) Total score of the Positive And Negative Syndrome Scale (PANSS) ≤ 60, indicating a stable stage of the condition. (d) Education level above primary school, normal intelligence, and ability to independently complete the scale assessment. (e) Antipsychotics administered at a stable therapeutic dose. Exclusion Criteria: (a) Individuals with religious beliefs. (b) Those suffering from other mental diseases (excluded based on ICD-10 Diagnostic Criteria). (c) Individuals with long-term meditation experience. (d) Those suffering from other brain diseases.

### Sample size and sampling procedure

The sample size was calculated using GPower 3.1 software, with a preliminary experiment effect size (d) of 0.432. Power analysis indicated that 158 participants would yield 85% statistical power at a two-sided α of 0.05 and effect size d = 0.432. Accounting for a 10% dropout rate, the planned total sample size was 174 participants. Randomization was conducted using Research Randomizer Version 4.0 (http://www.randomizer.org/), generating a list of random numbers for participant allocation to the experimental and control groups in a 1:1 ratio. The third author generated the random allocation sequence, and the first author enrolled and assigned participants to the respective groups.

### Measures

The assessments before and after the intervention were conducted by assessors unaware of the participants’ group assignments. Prior to the study, assessors underwent standardized training on evaluation methods and questionnaire comprehension. The questionnaire included: (a) Personal Information Questionnaire: This gathered information such as age, sex, marital status, duration of disease, first episode status, education, number of hospitalizations, family history of mental illness, Positive And Negative Syndrome Scale (PANSS) score, and length of hospital stay. (b) Connor-Davidson Resilience Scale (CD-RISC): The CD-RISC, a 25-item questionnaire, gauges psychological resilience across three factors: Toughness, Strength, and Optimism. Each item is scored from 0 (never or very rarely true) to 4 (very often or always true). The CD-RISC demonstrates robust construct validity and reliability (Cronbach α = 0.89–0.90) and is widely employed in clinical psychiatric settings [25, 26]. (c) Internalized Stigma of Mental Illness Scale (ISMI): The ISMI, comprising 29 items, assesses self-stigma among individuals with psychiatric disorders. It covers five factors: Alienation, Stereotype Endorsement, Perceived Discrimination, Social Withdrawal, and Stigma Resistance. ISMI exhibits strong construct validity and reliability (Cronbach α = 0.90–0.92) and is extensively used in clinical psychiatric settings [27]. (d) Rosenberg Self-Esteem Scale (RSES): The RSES, a 10-item questionnaire, measures self-esteem using a Likert point scale with four response choices (1 = completely agree; 4 = completely disagree). Total scores range from 10 to 40, with higher scores indicating elevated levels of individual self-esteem. The RSES demonstrates sound construct validity and reliability (Cronbach α = 0.90) and is widely employed in clinical psychiatric settings [27, 28].

### Interventions

Both the experimental and control groups received standard concomitant treatments, which primarily consisted of drug therapy, routine rehabilitation nursing activities, and health education. In addition to these standard interventions, the experimental group underwent a Mixed-mode Mindfulness-Based Intervention three times a week, with each session lasting 60–90 min. The Mixed-mode Mindfulness-Based Cognitive Therapy (M-MBCT) course adopted a structured approach, combining “face-to-face Group MBCT course (4 weeks)” with “self-help MBCT course (4 weeks).” The face-to-face group therapy sessions occurred in the first, third, fifth, and seventh weeks, each accommodating 10–12 patients. Professional psychotherapists facilitated these sessions, providing group mindfulness-based therapy and addressing any participant queries. During the second, fourth, sixth, and eighth weeks, the self-help mindfulness-based course was implemented. This phase utilized audio guidance and video teaching as primary instructional methods, with ongoing collection of patient feedback and completion levels. Patients encountering challenges or expressing dissatisfaction received timely communication to resolve issues and ensure the efficacy and compliance of the self-help mindfulness-based course. Refer to Table 1 for the specific schedule of the Mixed-Mode Mindfulness-Based Cognitive Therapy course.

**Table 1:** Mixed-mode mindfulness-based cognitive therapy course schedule

Time	Mode	Content
First week	Group therapy	Body scan; Mindfulness breathing; Mindfulness of daily life; Feeling mindfulness from the inside out; Improve concentration;
Second week	Self-help therapy	Body scan; Mindfulness yoga; Get out of ruminant thinking ; Stop automatic navigation;
Third week	Group therapy	Body scan; Sitting meditation; Mindfulness yoga; Thoughts are not equal to reality; Live in the moment;ABC Theory of Emotion;
Fourth week	Self-help therapy	Body scan; Mindfulness yoga; Sitting meditation; Awareness of thoughts and emotions; Awareness of pleasant and unpleasant events;
Fifth week	Group therapy	Body scan; Sitting meditation; Mindfulness yoga; Accept negative emotions and refuse to escape;
Sixth week	Self-help therapy	Body scan; Sitting meditation; Mindfulness breathing; How to take better care of yourself;
Seventh week	Group therapy	Body scan; Sitting meditation; Mindfulness yoga; Explore how to integrate mindfulness into life;
Eighth week	Self-help therapy	Body scan; Sitting meditation, Mindfulness breathing; Explore a sustainable mindfulness practice mode;

### Statistical analysis

Descriptive analysis was employed to compute the scores for each measurement scale. To compare demographic data between the intervention and control groups at baseline, a one-way ANOVA was conducted. Dependent-sample t-tests were utilized to compare pre/post intervention mean scores of the scales between the intervention and control groups. Paired t-tests were performed to analyze within-group differences in scores. Independent-samples t-tests were employed to compare mean scores of the scales between participants in the control and experimental groups. Pearson’s correlation coefficient was calculated for correlation analysis. Exploration of relevant factors influencing stigma involved multiple linear regression analysis. Data analyses were conducted using the SPSS statistical package, version 19.0 (SPSS Inc., Chicago, IL, USA). All statistical tests were two-sided, and the level of significance was set at *P* < 0.05.

## Results

**Fig. 1:**
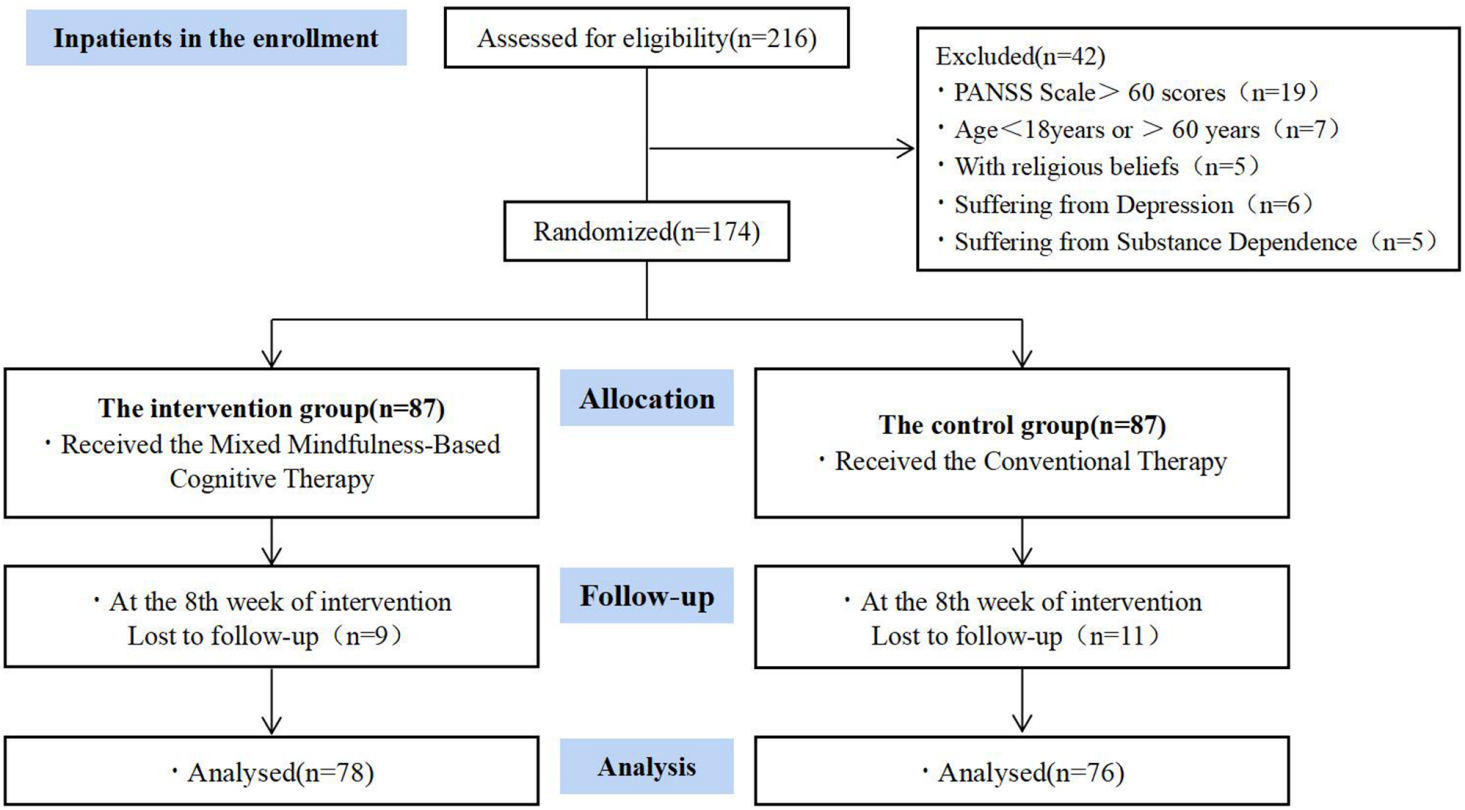
Recruitment and participant flow

Out of 216 patients with schizophrenia initially screened for eligibility, 174 patients were found eligible and provided their consent for participation, subsequently receiving randomized allocation. At the conclusion of the intervention, assessments were completed by 154 participants, with 9 participants lost from the experimental group and 11 from the control group. Table 2 displays the demographic characteristics of the participants, revealing no significant differences in baseline data between the two groups (*P* > 0.05). Refer to Fig. 1 for a visual representation of participant enrollment and dropouts.

**Table 2:** Demographic characteristics of participants

Variable	Experimental group (n = 78)	Control group (n = 76)	*t/x2*	*P*
Age (years), Mean (SD)	45.67 (9.03)	45.97 (8.67)	-0.215	0.830
Disease duration (years), Mean (SD)	20.81 (9.72)	19.41 (9.26)	0.915	0.362
Married, n (%)			0.475	0.924
Unmarried	33 (42.31)	35 (46.05)		
Married	16 (20.51)	15 (19.74)		
Divorced	27 (34.62)	25 (32.89)		
Widowed	2 (2.56)	1 (1.32)		
Education (years), Mean (SD)	11.27 (2.84)	10.78 (2.71)	1.103	0.272
Sex, n (%)			0.025	0.874
Male	38 (48.72)	38 (50.00)		
Female	40 (51.28)	38 (50.00)		
Number of hospitalizations, Mean (SD)	15.32 (13.71)	15.62 (11.67)	-0.145	0.885
First-episode schizophrenia, n (%)			0.178	0.673
Yes	3 (3.85)	4 (5.26)		
No	75 (96.15)	72 (94.74)		
Score on the Positive And Negative Syndrome Scale, Mean (SD)	48.53 (6.66)	48.75 (6.85)	-0.206	0.837
Family history of mental illness, n (%)			1.179	0.278
Yes	13 (16.67)	18 (23.68)		
No	65 (83.33)	58 (76.32)		

SD = standard deviation. *x*^*2*^ = Pearson Chi-square test, *t* = Independent t-test. ^*^Statistically significant *p*-value at ≤ 0.05

No significant differences were observed in the scores of the CD-RISC scale, ISMI scale, and RSES scale between the control group and the experimental group before the intervention (*P* > 0.05). Following 8 weeks of M-MBCT intervention, noteworthy differences emerged. The total score of the CD-RISC scale and scores of Strength and Optimism in the experimental group were significantly higher than those in the control group (*P* < 0.05). Moreover, the total score of the ISMI scale and scores of Stereotype Endorsement, Perceived Discrimination, Social Withdrawal, and Stigma Resistance in the experimental group were significantly lower than those in the control group (*P* < 0.05). Additionally, the total score of the RSES scale in the experimental group was significantly higher than that in the control group (*P* < 0.05). For detailed comparisons before and after the intervention, please refer to Table 3.

**Table 3:** Comparison of the two groups before and after intervention

Variable	Group	N	Pre-intervention Mean (SD)	*t*	*P*	Post-intervention Mean (SD)	*t*	*P*
CD-RISC	EG	78	49.15 (8.85)	-0.573	0.568	54.96 (10.39)	3.192	0.002^**^
	CG	76	49.99 (9.20)			49.87 (9.36)		
Toughness	EG	78	26.14 (4.86)	-0.932	0.353	28.63 (6.22)	1.547	0.124
	CG	76	26.91 (5.34)			27.12 (5.88)		
Strength	EG	78	14.91 (4.07)	0.043	0.966	17.00 (4.48)	3.301	0.001^**^
	CG	76	14.88 (4.17)			14.80 (3.74)		
Optimism	EG	78	8.10 (2.38)	-0.241	0.810	9.33 (2.84)	3.314	0.001^**^
	CG	76	8.20 (2.50)			7.95 (2.32)		
ISMI	EG	78	66.17 (9.41)	-0.488	0.626	60.38 (10.19)	-3.932	0.000^***^
	CG	76	66.95 (10.42)			66.97 (10.60)		
Alienation	EG	78	15.26 (3.12)	0.971	0.333	14.23 (3.47)	-0.705	0.482
	CG	76	14.70 (3.97)			14.66 (4.03)		
Stereotype Endorsement	EG	78	14.51 (3.60)	0.276	0.783	13.26 (3.54)	-2.108	0.037
	CG	76	14.34 (4.06)			14.51 (3.85)		
Perceived Discrimination	EG	78	9.03 (2.11)	-1.533	0.127	7.81 (1.91)	-5.388	0.000^***^
	CG	76	9.61 (2.57)			9.78 (2.57)		
Social Withdrawal	EG	78	13.91 (3.79)	-1.807	0.073	12.91 (3.76)	-3.280	0.001^**^
	CG	76	14.97 (3.50)			14.78 (3.28)		
Stigma Resistance	EG	78	13.46 (3.19)	0.261	0.794	12.18 (3.26)	-2.035	0.044^*^
	CG	76	13.33 (3.11)			13.25 (3.27)		
RSES	EG	78	22.51 (4.02)	-0.236	0.814	26.18 (4.06)	4.995	0.000^***^
	CG	76	22.67 (4.31)			22.91 (4.07)		

CD-RISC = Condor-Davidson Resilience Scale, ISMI = Internalized Stigma of Mental Illness Scale, RSES = Rosenberg Self-Esteem Scale. EG = the experimental group, CG = the control group. SD = standard deviation. *t* = independent t-test, ^*^Statistically significant *p*-value at ≤ 0.05, ^**^Statistically significant *p*-value at ≤ 0.01, ^**^Statistically significant *p*-value at ≤ 0.001.

In the experimental group, significant within-group improvements were observed after the M-MBCT intervention. Specifically, the total score of the CD-RISC scale and scores of Toughness, Strength, and Optimism were significantly higher after the intervention compared to before (*P* < 0.05). Furthermore, the total score of the ISMI scale and scores of Alienation, Stereotype Endorsement, Perceived Discrimination, Social Withdrawal, and Stigma Resistance were significantly lower after the intervention than before (*P* < 0.05). Additionally, the total score of the RSES scale was significantly higher after the intervention compared to before (*P* < 0.05). In contrast, in the control group, no significant differences were observed in the scores of each scale before and after the intervention (*P* > 0.05). For detailed within-group comparisons before and after the intervention, please refer to Table 4.

**Table 4:** Within group comparisons for each group before and after the intervention

Variable	Before or after intervention	EG(n = 78)Mean (SD)	*t*	*P*	CG(n = 76)Mean (SD)	*t*	*P*
CD-RISC	Before	49.15 (8.85)	-6.968	0.000^***^	49.99 ( 9.20)	0.365	0.716
	After	54.96 (10.39)			49.87 (9.36)		
Toughness	Before	26.14 (4.86)	-4.632	0.000^***^	26.91 (5.34)	-1.203	0.233
	After	28.63 (6.22)			27.12 (5.88)		
Strength	Before	14.91 (4.07)	-5.496	0.000^***^	14.88 (4.17)	0.428	0.670
	After	17.00 (4.48)			14.80 (3.74)		
Optimism	Before	8.10 ± 2.38)	-4.579	0.000^***^	8.20 (2.50)	1.530	0.130
	After	9.33 (2.84)			7.95 (2.32)		
ISMI	Before	66.17 (9.41)	5.257	0.000^***^	66.95 (10.42)	-0.076	0.940
	After	60.38 (10.19)			66.97 (10.60)		
Alienation	Before	15.26 (3.12)	2.756	0.007^**^	14.70 (3.97)	0.267	0.790
	After	14.23 (3.47)			14.66 (4.03)		
Stereotype Endorsement	Before	14.51 (3.60)	3.161	0.002^**^	14.34 (4.06)	-1.052	0.296
	After	13.26 (3.54)			14.51 (3.85)		
Perceived Discrimination	Before	9.03 (2.11)	5.752	0.000^***^	9.61 (2.57)	-1.849	0.068
	After	7.81 (1.91)			9.78 (2.57)		
Social Withdrawal	Before	13.91 (3.79)	2.509	0.014^*^	14.97 (3.50)	1.806	0.075
	After	12.91 (3.76)			14.77 (3.28)		
Stigma Resistance	Before	13.46 (3.19)	3.433	0.001^**^		0.652	0.940
	After	12.18 (3.26)			13.25 (3.27)		
RSES	Before	22.51 (4.02)	-6.873	0.000^***^	22.67 (4.31)	-0.806	0.423
	After	26.18 (4.06)			22.91 (4.07)		

CD-RISC = Condor-Davidson Resilience Scale, ISMI = Internalized Stigma of Mental Illness Scale, RSES = Rosenberg Self-Esteem Scale. EG = the experimental group, CG = the control group. SD = standard deviation. *t* = independent t-test. ^*^ Statistically significant *p*-value at ≤ 0.05, ^**^ Statistically significant *p*-value at ≤ 0.01, ^***^ Statistically significant *p*-value at ≤ 0.001

In the fully enrolled patients with schizophrenia, a multiple linear regression analysis was conducted to examine factors related to stigma. The total score of stigma was considered the dependent variable, and the following independent variables were included: sex (male = 1, female = 2), age, marital status (unmarried = 1, married = 2, divorced = 3, widowed = 4), education, first-episode schizophrenia (Yes = 1, No = 2), family history of mental illness (Yes = 1, No = 2), disease course, and number of hospitalizations. The multiple linear regression analysis revealed that age, education, and family history of mental illness were significant factors related to stigma (*P* < 0.05). For detailed information on the multiple linear regression analysis and the factors related to stigma, please refer to Table 5.

**Table 5:** Multiple linear regression analysis of factors related to stigma of patients with schizophrenia

Variable	B	Standard error	*β*	*t*	*P*
Age	-0.571	0.105	-0.509	-5.459	0.000^***^
Disease duration	-1.718	0.092	-0.019	-0.21	0.834
Married	1.256	0.818	0.118	1.536	0.127
Education	0.699	0.242	0.197	2.887	0.004^**^
Sex	-1.718	1.355	-0.087	-1.268	0.207
Number of hospitalizations	0.011	0.057	0.014	0.197	0.844
First-episode schizophrenia	-4.334	3.356	-0.092	-1.291	0.199
Family history of mental illness	-5.501	1.627	-0.224	-3.382	0.001^**^

B = regression coefficient, *β =* standardized regression coefficient. ^**^ Statistically significant p-value at ≤ 0.01, ^***^ Statistically significant p-value at ≤ 0.001

In the experimental group, correlation analysis was conducted to examine relationships between variables. The score reduction of CD-RISC (intervention after reduction intervention before) was found to be negatively correlated with the score reduction of ISMI (*r* = -0.863, 95%CI: -0.956 - -0.722, *P* < 0.001). Additionally, the score reduction of ISMI was negatively correlated with the score reduction of RSES (*r* = -0.782, 95%CI: -0.874 - -0.631, *P* < 0.001).

## Discussion

Psychological resilience functions as a protective mechanism, enabling individuals to confront negative stressors by reshaping their relationship with the environment and dynamically enhancing adaptability [11, 12]. This study affirms that Mixed-mode Mindfulness-Based Cognitive Therapy (M-MBCT) effectively elevates psychological resilience levels in patients with schizophrenia. Consistent with prior research, Mindfulness-Based Cognitive Therapy (MBCT) has shown efficacy in improving resilience in patients with chronic diseases and mental disorders [29]. Possible reasons for this positive impact include: (a) Neurological Changes: Mindfulness-based therapy may enhance the activity and functional connectivity of pressure-regulating regions in the prefrontal cortex, increase coupling of regulatory regions, and improve connectivity within the executive control network. These changes potentially enhance stress resistance in patients [30]. (b) Neuroplasticity Effects: Mindfulness-based therapy may induce neuroplasticity changes in key areas related to emotional response, body consciousness, self-consciousness, and emotion regulation. These changes contribute to reducing stress responses and enhancing individuals’ ability to cope with negative events [31]. (c) Emotional Regulation: MBCT may effectively reduce negative emotions stemming from rumination, addressing associated behaviors or thoughts. This improvement in emotional regulation enhances patients’ adaptive capacity in response to environmental and emotional changes [32].

Given the increasing recognition of internalized stigma as a crucial indicator for measuring recovery in patients with schizophrenia, attention to stigma levels has become paramount [33]. In the Chinese context, where cultural connotations of Confucianism emphasize ‘face’ and perpetuate derogatory beliefs about mental illness, stigma associated with schizophrenia is uniquely manifested, contributing to elevated levels of stigma among Chinese individuals [34]. This study’s findings reveal that M-MBCT is effective in reducing stigma among patients with schizophrenia, encompassing Stereotype Endorsement, Stigma Resistance, Social Withdrawal, and Perceived Discrimination. The efficacy of M-MBCT in improving stigma resistance and reducing stereotype endorsement, social withdrawal, and perceived discrimination aligns with previous studies demonstrating the stigma-reducing potential of MBCT [20]. Possible reasons for the stigma reduction observed in this study include: (a) Cognitive Functioning Enhancement: Mindfulness therapy may positively impact cognitive functioning by enhancing self and metacognition, challenging misconceptions about mental illness, and fostering acceptance of the reality of the condition. This, in turn, empowers patients to confront stigma more effectively, boosting confidence and treatment adherence [35, 36]. (b) Attention Control: Mindfulness therapy might enhance patients’ attentional control, fostering a positive understanding of the surrounding environment and mitigating the negative impact of social opinions. This expansion of positive experiences in life may effectively reduce stigma arising from social bias and discrimination [37–38]. (c) Stigma-Coping Orientation: MBCT may improve patients’ stigma-coping orientation, evidenced by changes in factors like secrecy, withdrawal, education, and challenge. Patients, post-intervention, are more inclined to accept and share their experiences related to mental illness, leading to reduced secrecy and withdrawal behaviors [20]. (d) Resilience Improvement: The study found a negative correlation between the reduction of psychological resilience (CD-RISC) and the reduction of stigma (ISMI), indicating that improving resilience is associated with reduced stigma. This aligns with previous research highlighting the benefits of psychological resilience in reducing stigma among patients [14]. (e) Internal Feelings Transformation: Mindfulness therapy may alter internal feelings of patients, boosting confidence in disease recovery, enhancing happiness, and improving social functioning. This transformation contributes to alleviating the inferiority complex associated with schizophrenia and increasing patients’ confidence in social activities [39].

The study demonstrates that Mixed-mode Mindfulness-Based Cognitive Therapy (M-MBCT) effectively improves the self-esteem levels of patients with schizophrenia. This improvement can be attributed not only to the specific mechanisms of mindfulness-based therapy but also to the concurrent enhancement of resilience and reduction of stigma. Internalized stigma, as highlighted in previous research, contributes to the social isolation of patients with schizophrenia, leading to diminished self-esteem and impeding rehabilitation [4–6]. The current study reveals a negative correlation between reductions in ISMI scores and RSES scores, indicating that reducing stigma is associated with improved self-esteem. Furthermore, enhancing resilience also positively contributes to improved self-esteem [14].

The study identifies age, education, and family history of mental illness as potential influencing factors for stigma in patients with schizophrenia. Possible explanations include: (a) Age Differences: Younger patients are often at critical stages of learning or early career development, and a diagnosis of schizophrenia may impact their marriage prospects, educational opportunities, and employment options. In contrast, older patients tend to have more stable work and family environments, along with better social support systems. (b) Educational Background: Patients with higher levels of education may possess better jobs, reputations, and social statuses. Consequently, a diagnosis of schizophrenia could result in the potential loss of these advantages. (c) Family History: Patients with a family history of mental illness might have a deeper understanding of the short-term and long-term adverse effects of schizophrenia on both individuals and families.

Numerous studies have consistently demonstrated the effectiveness of MBCT for various common mental, physical, and social health conditions across diverse populations [37, 39]. Moreover, MBCT is considered relatively safe [37]. However, the application of MBCT is constrained by factors such as cost, time commitment from professional therapists, and the stigma associated with seeking treatment multiple times [22, 37, 40]. M-MBCT presents a promising solution by adopting a blended approach, combining face-to-face group therapy courses with a self-help component delivered through audio guidance and video teaching. In comparison to traditional MBCT, M-MBCT offers several advantages, including: (a) Enhanced Autonomy: M-MBCT provides individuals with a certain degree of autonomy in managing their mental health. (b) Reduced Time Investment: The approach reduces the time investment required from professional therapists. (c) Improved Convenience: M-MBCT offers increased convenience for participants. (d) Reduced Treatment Costs: M-MBCT is associated with lower treatment costs. (e) Stigma Reduction: The blended approach somewhat mitigates the stigma related to multiple treatment sessions. Moreover, when compared to self-help MBCT, M-MBCT effectively addresses limitations such as the lack of supervision, therapist involvement, and absence of group discussions. Consequently, M-MBCT holds significant potential for widespread implementation among individuals diagnosed with schizophrenia.

## Limitations

Despite the valuable insights gained, the study has limitations, including a small sample size, recruitment from a single location, and a lack of long-term follow-up. Future research should consider large sample sizes, multi-center research, and extended follow-up periods. Additionally, although no significant differences were observed between the experimental and control groups at baseline, the study did not collect information on prescription drug dosages and related symptoms. Future studies should incorporate these factors for a more comprehensive evaluation.

## Conclusions

The research findings provide support for the positive effects of M-MBCT on individuals diagnosed with schizophrenia. M-MBCT demonstrates effective enhancement in resilience levels, self-esteem, and reduction in stigma among patients with schizophrenia. The project holds potential for widespread implementation within the population of individuals diagnosed with schizophrenia.

## Data Availability

The datasets utilized and analyzed during the current study are available from the corresponding author upon request.

## Abbreviations

MBCT: Mindfulness-Based Cognitive Therapy
M-MBCT: Mixed-mode Mindfulness-Based Cognitive Therapy
CD-RISC: Connor-Davidson Resilience Scale
ISMI: Internalized Stigma of Mental Illness Scale
RSES: Rosenberg Self-Esteem Scale
MBSR: Mindfulness-Based Stress Reduction
PANSS: Positive And Negative Syndrome Scale
